# Errata

**DOI:** 10.36660/abc.20210642

**Published:** 2021-08-09

**Edição de Maio de 2019, vol. 112 (5), págs. 649-705**

Na “Atualização das Diretrizes em Cardiogeriatria da Sociedade Brasileira de Cardiologia – 2019”, com número de DOI: https://doi.org/10.5935/abc.20190086, publicado no periódico Arquivos Brasileiros de Cardiologia, 112(5):649-705, foi incluído o nome do autor Felipe Costa Fuchs na pág. 649, entre os autores do capítulo 6; na pág. 650, entre os autores da atualização; e na pág. 652, na declaração de conflito de interesse.

**Edição de Maio de 2020, vol. 114 (5), págs. 943-987**

Na versão em inglês da “Diretriz Brasileira de Reabilitação Cardiovascular – 2020”, com número de DOI: https://doi.org/10.36660/abc.20200407, publicado no periódico Arquivos Brasileiros de Cardiologia, 114(5):943-987, corrigir o valor “50–85%” na linha 4, coluna 2, para “50–80%”, conforme versão português do documento.

