# Supplementary material for: Errata
Source: Arq Bras Cardiol. 2021 Aug 9;117(2):423. [Article in Portuguese] doi: 10.36660/abc.20210642 (PMC8395795; doi:10.36660/abc.20210642)
Supplement: Translation

# Translation: Erratum

**May 2019 Issue, vol. 112 (5), pages 649-705**

In the “Updated Geriatric Cardiology Guidelines of the Brazilian Society of Cardiology – 2019”, with DOI: https://doi.org/10.5935/abc.20190086, published in the journal Arquivos Brasileiros de Cardiologia, the name of the author Felipe Costa Fuchs was included on page 649, in the authors of chapter 6; on page 650, in the update authors; and on page 652, in the declaration of potential conflict of interest.

**May 2020 Issue, vol. 114 (5), pages 943-987**

In the English version of the “Updated Geriatric Cardiology Guidelines of the Brazilian Society of Cardiology – 2019”, with DOI: https://doi.org/10.36660/abc.20200407, published in the journal Arquivos Brasileiros de Cardiologia, 114(5):943-987, correct the value “50–85%” in line 4, column 2, to “50–80%”, according to the Portuguese version of the document.
